# Survival relative to new and ancestral host plants, phytoplasma infection, and genetic constitution in host races of a polyphagous insect disease vector

**DOI:** 10.1002/ece3.1158

**Published:** 2014-07-15

**Authors:** Michael Maixner, Andreas Albert, Jes Johannesen

**Affiliations:** 1 Institute for Plant Protection in Fruit Crops and Viticulture, Julius Kühn-Institut (JKI), Federal Research Institute for Cultivated Plants76833, Siebeldingen, Germany; 2 Institute of Zoology, University of Mainz55128, Mainz, Germany

**Keywords:** Gene–behavior interaction, host-race evolution, *Hyalesthes obsoletus*, microsatellites, stolbur phytoplasma, tritrophic interaction

## Abstract

Dissemination of vectorborne diseases depends strongly on the vector's host range and the pathogen's reservoir range. Because vectors interact with pathogens, the direction and strength of a vector's host shift is vital for understanding epidemiology and is embedded in the framework of ecological specialization. This study investigates survival in host-race evolution of a polyphagous insect disease vector, *Hyalesthes obsoletus*, whether survival is related to the direction of the host shift (from field bindweed to stinging nettle), the interaction with plant-specific strains of obligate vectored pathogens/symbionts (stolbur phytoplasma), and whether survival is related to genetic differentiation between the host races. We used a twice repeated, identical nested experimental design to study survival of the vector on alternative hosts and relative to infection status. Survival was tested with Kaplan–Meier analyses, while genetic differentiation between vector populations was quantified with microsatellite allele frequencies. We found significant direct effects of host plant (reduced survival on wrong hosts) and sex (males survive longer than females) in both host races and relative effects of host (nettle animals more affected than bindweed animals) and sex (males more affected than females). Survival of bindweed animals was significantly higher on symptomatic than nonsymptomatic field bindweed, but in the second experiment only. Infection potentially had a positive effect on survival in nettle animals but due to low infection rates the results remain suggestive. Genetic differentiation was not related to survival. Greater negative plant-transfer effect but no negative effect of stolbur in the derived host race suggests preadaptation to the new pathogen/symbiont strain before strong diversifying selection during the specialization process. Physiological maladaptation or failure to accept the ancestral plant will have similar consequences, namely positive assortative mating within host races and a reduction in the likelihood of oviposition on the alternative plant and thus the acquisition of alternative stolbur strains.

## Introduction

The epidemiology of insect-vectorborne plant diseases is based on the tritrophic interaction between the vector, the pathogen, and the reservoir host for pathogen proliferation, which is not necessarily a diseased host. If pathogens are effectively neutral symbionts in the insect vector (but not in the diseased end plant host), infection potential including spillover to nonreservoir hosts will be influenced by the vector's host range for pathogen acquisition and by the potentially wider feeding range (pathogen transmission). Narrow host and feeding ranges may lead to specialized transmission cycles, whereas polyphagous vectors may introduce different pathogens to diverse end hosts (Maixner 2011; Mannelli et al. 2012).

A key question in the study of vectorborne plant diseases is whether the plant pathogen interacts with the vector to influence the vector's specialization to and preference for plants (Biere and Tack 2013) and thus disease dissemination. Benefits for the vector include increased longevity and fecundity (Beanland et al. 2000) and adult survival (Ebbert and Nault 2001; Belliure et al. 2005). Interactions may be complex and vary relative to temperature and host plant (Stumpf and Kennedy 2005), and the plant pathogen can manipulate the vector to prefer infected reservoir hosts (Mayer et al. 2008). Elliott et al. (2003) showed that parasites in a patchy environment might become benign toward the vector when it is the more mobile species, free parasites rarely disperse and parasite strains do not compete. If vectors feed on new hosts with new “maladapted” strains or experience competition between different strains, virulence toward the vector may increase. Hence, new host preferences of the vector may change or introduce new dissemination pathways, including the emergence of new diseases.

The evolution of new host preferences is probably the major route to intraspecific divergence and ultimately speciation in phytophagous insects (Dres and Mallet 2002). When populations cannot simultaneously adapt to a new and an old host, insect–plant (parasite-host)-associated fitness trade-offs may evolve (Rausher 1984; Via 1990), leading to partially reproductively isolated insect populations called host races (Diehl and Bush 1984). However, as noted by Singer et al. (1989, 1992), host shifts in polyphagous insects may include shifts to plants that are potentially suitable but go unused due to inapt environmental conditions. When conditions improve, plants lower in the preference hierarchy may again be included in the diet (Hodkinson 1997). In this case, the evolution of host races requires permanent differential utilization and not merely a temporary shift. Quantifying which and/or how traits evolve and in which succession thus not only sheds light on diversification modes but also have implications for pest management of vectorborne diseases by identifying ecological or behavioral differences among vector populations (Barrett et al. 2008).

The planthopper *Hyalesthes obsoletus* (Signoret, 1865) (Hemiptera: Cixiidae) is the main vector of *Candidatus* Phytoplasma solani (stolbur phytoplasma) (Quaglino et al. 2013). Phytoplasma are wall-less, nonhelical prokaryotes that colonize plant phloem and depend on phloem-feeding insect vectors (leafhoppers, planthoppers, and psyllids) for transmission (Weintraub and Beanland 2006). Stolbur phytoplasma are responsible for several emerging yellows diseases in Europe of which the “bois noir” disease has become a severe disease of grapevine. Stolbur phytoplasma are divided into two genetically distinct strains, tuf-a and tuf-b (Langer and Maixner 2004). The reservoir plant of the tuf-a strain is, as far as we know, exclusively stinging nettle (*Urtica dioica* L.) (Langer and Maixner 2004; Bressan et al. 2007), whereas the tuf-b strain is associated with a wider range of herbaceous hosts, field bindweed (*Convolvulus arvensis* L.) being the major one (Credi et al. 2006; Ember et al. 2011). Stinging nettle and field bindweed are also the principal hosts of the vector. Hence, being both host and reservoirs for the vector and stolbur phytoplasma, the two plants significantly influence the epidemiology of stolbur-induced yellows diseases. In the case of the grapevine disease bois noir, the herbaceous plants solely determine the epidemiology as grapevine is a dead-end host for stolbur phytoplasma.

*Hyalesthes obsoletus* is regarded as polyphagous with an innate potential to use both stinging nettle and field bindweed in its core Mediterranean distribution range, as mtDNA and microsatellite marker distributions also suggest (Johannesen et al. 2012; Imo et al. 2013). At the northern range border in southwest Germany and northeast France (Alsace), field bindweed was the only known host until about 25 years ago, when the rare occurrence of *H. obsoletus* was restricted to climatically favorable river valleys associated with viticulture. Since then, in the northern range, stinging nettle has become a preferred plant, population sizes have increased dramatically and the specific stinging nettle-stolbur tuf-a strain is now a severe cause of infection pressure on grapevine. Genetic analyses support a sympatric host shift of *H. obsoletus* from field bindweed to stinging nettle over the past, perhaps <50 years in the northern range, and the evolution of two genetically distinct host-associated populations that can be regarded as host races (Imo et al. 2013). The sudden emergence of the tuf-a strain in the northern stinging nettle-associated host race, however, likely proceeded via an independent recent demographic expansion of the vector in Western Europe where plant-unspecific vectors using stinging nettle introduced the tuf-a strain into the northern stinging nettle host-race cycle (Johannesen et al. 2012). Hence, two independent processes likely determined the dissemination of the obligate vectored and specialized tuf-a stolbur in different regions of Western Europe.

The aim of the present study is set within the frameworks of two fields studying insect diversification that have epidemiological and applied significance for the specificity of insect vector-borne plant diseases: (1) adaptations in host-race evolution and (2) the consequences of pathogen/symbiont infection for the diversification process. Specifically, we tested (1) survival on alternative plants as signs of physiological adaptations and/or acceptance hierarchies in host-race evolution, (2) the relative strength of the interaction in the host races, (3) the interaction with stolbur infection on this process and (4) whether survival can be related to the genetic differentiation between the two host races as seen in diversification at microsatellite loci (Imo et al. 2013). Because we know the direction of the host shift, from field bindweed to stinging nettle, we further address (5) whether the relative outcome of survival is related to the direction of the host shift. In a previous study, Kessler et al. (2011), found that animals caught on stinging nettle from a population that could be genetically differentiated relative to host plant (Maniyar et al. 2013) survived longer on stinging nettle but they did not analyze reciprocal effects with field bindweed associated insects or the effect of stolbur infection on adult survival. In a preliminary conference report, Johannesen et al. (2011) showed evidence for differential survival of females on alternative plants. There is no clear prediction as to which population will be affected the most, but assuming that individuals of populations associated with stinging nettle (hereafter nettle animals) are derived and highly specialized for this plant host, and individuals of populations associated with field bindweed (hereafter bindweed animals) are ancestral and potentially more polyphagous (despite genetically distinct due to diversification of nettle animals), one may speculate that a host-plant effect will be most pronounced in nettle animals.

## Material and Methods

### Host plants

The two host plants, field bindweed and stinging nettle, are common plants in Europe and in the study area, southwest Germany. Field bindweed infected with stolbur display specific disease symptoms (stunted growth, cupped, and yellow leaves), whereas infected stinging nettle are symptomless. Young plants of symptomatic and nonsymptomatic field bindweed were collected before the experiments in and around vineyards, where no herbicides had been applied, to rule out yellowing symptoms caused by herbicides. They were collected in the same area as the insects but not in the same plots. The plants were grown in pots to produce shoots of similar quality for the experiments. Stinging nettle was grown from seed in the greenhouse. After termination of the experiments, one symptomatic field bindweed leaf from each cage was tested for stolbur with specific PCR. These tests failed to detect stolbur. This negative result is puzzling but may have been caused by the experimental conditions where field bindweed shoots were held in water-only vials, which can reduce the phytoplasma titer. We therefore refer to aberrant field bindweed as symptomatic rather than infected. These results are suggestive of stolbur interaction but not direct proof thereof.

### Sampling and survival experiment

All experimental *H. obsoletus* were caught in the field on field bindweed or stinging nettle with sweep nets, transferred to 100-mL bottles with exhausters and separated by sex on the day they were caught. Due to nymphal development on roots of host plants, the univoltine *H. obsoletus* is extremely difficult to breed in the laboratory. In the study area, adult emergence of each host race can be predicted with temperature-sum models; bindweed animals emerge about 2 weeks before nettle animals (Maixner and Johannesen in press[Bibr b38]). Hence, adults of each host race can be caught at standardized timepoints (day) within their flight period, which enables the sampling of cohorts with similar mean ages. Moreover, as individuals of the two host-race populations were collected at the same timepoints of their respective flight periods, their age was also comparable. Each host race was caught in two cohorts for repeated analyses. Bindweed animals were caught on the 22.06.2010 and 30.06.2010 at Bernkastel-Kues (Moselle), and nettle animals were caught on the 12.07.2010 and 20.07.2010 at Kesten (Moselle). The first sampling date (cohort) for each host race corresponds to 2 weeks into the flight period. The two collection sites were situated 7 km apart.

**Figure 1:**
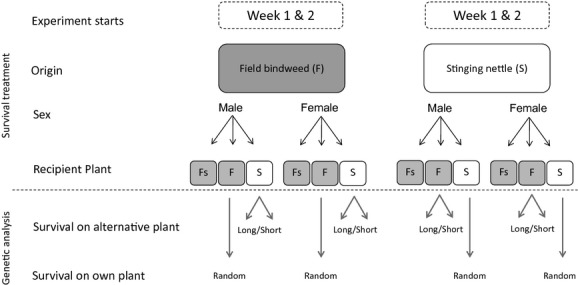
Experimental design for survival study and genetic-survival correlations. Each host-race population was exposed to identical treatments (stinging nettle (S), symptomatic (Fs) and nonsymptomatic (F) field bindweed) in two experimental weeks (1 and 2). Males and females were tested separately. Genetic associations between survival and genotypes were studied by comparing the genetic membership of individuals surviving the shortest (Short) and the longest (Long) on alternative plants with individuals sampled randomly (Random) on their own host plants.

Survival was analyzed in twice-repeated experiments in each host race and based on cohorts from the sampling dates given above (referred to as week 1 and week 2). Each experiment started the day after collection and had three treatments (Fig. 1): (1) survival on stinging nettle, (2) survival on nonsymptomatic field bindweed and (3) survival on symptomatic field bindweed. Each treatment was performed separately for males and females. For each treatment and sex, five individuals were placed in each of 10 cages (lidded 500-ml plastic cups) containing a small plant shoot with 2–3 leaves of either field bindweed or stinging nettle. The shoots were immerged in water-filled vials sealed with foil to avoid evaporation. The cages were kept at constant temperature (25°C) at day/night regime of 16:8 h (day: 6 am–10 pm). The intended number of individuals in the study was 1200 = 2 host races × 2 experiments × 3 treatments × 2 sexes × (5 individuals × 10 cages). In total, however, we tested 1240 individuals due to the inclusion of more than 50 individuals in the first week of the field bindweed experiment. As 22 specimens were lost during the experiments, 1218 individuals were tested in total. All cages were checked on a daily basis, and individual survival was measured as the number of days surviving. All animals were tested for stolbur infection after termination of the experiments.

Due to the nested and interaction nature of the experimental design, we first tested whether sex and experimental week affected survival before analyzing the treatment (plant) effects between pairs of group variables. The effects of cages were not considered in the analyses as survival relative to sex and week across cages was the focus of interest. Differences in mean survival between group categories and treatments, and relative to stolbur infection were tested with Kaplan–Meier survival analysis (Log-Rank tests) (JMP 4.0.4; SAS Institute, Cary, North Carolina, USA). All Kaplan–Meier tests have 1 degree of freedom against a chi-squared distribution. Different infection rates between host races and between sexes within host races were tested with Fisher's exact tests using JMP.

### Stolbur phytoplasma detection

Stolbur phytoplasma were detected via PCR with specific primers using genomic DNA isolated from the experimental *H. obsoletus* and from the symptomatic field bindweed. DNA extraction from insects was performed using the High Pure PCR Template Preparation Kit (Roche Diagnostics, Mannheim, Germany) following the manufacturer′s protocol. DNA was extracted from field bindweed as described by Maixner et al. (1995). The extracted DNA was stored at −20°C until use. The detection of stolbur was carried out first by amplifying a partial sequence of the 16S rRNA gene with the primers rStol: 5-AGA TGT GAC CTA TTT TGG TGG-3 and fStol: GCC ATC ATT AAG TTG GGG A (Maixner et al. 1995). Stolbur positive individuals were then checked for tuf-type with nested PCR using the primers and protocol in Schneider et al. (1997). The tuf amplification product was cut with the restriction enzyme *Hpa*II to reveal tuf-a and tuf-b infections using the protocol in the study of Langer and Maixner (2004).

### Genetic differentiation

We used seven microsatellite loci (Imo et al. 2011) to confirm (1) genetic differentiation among nettle and bindweed populations, (2) to exclude the possibility that analyzed individuals came from the “wrong” host plant, and (3) to test for associations between alleles/loci and survival on alternative plants. All loci show stronger host race than local geographic differentiation (Imo et al. 2013). While the former two give evidence for genetically differentiated samples of *H. obsoletus*, the latter association was analyzed for indications of linkage between loci/alleles and survival. The genetic tests were based on individuals caught in the first week of each host plant.

In the first and second analyses, we tested whether the experimental host populations were genetically differentiated and assessed the likelihood of experimenting with “wrong” individuals, that is migrating bindweed animals caught on stinging nettle or vice versa. In these tests, we also checked whether the two experimental populations, which were collected 7 km apart, were differentiated genetically by geographic distance rather than host plant. To confirm plant-related differentiation, we compared the two random host-plant samples (*H. obsoletus* collected on own host plants) with a syntopic bindweed and nettle population situated 100 km away (Bacharach) (Imo et al. 2011). We tested differentiation with pairwise genetic variance, *F*_ST_, among the four populations (Arlequin; Excoffier and Lischer 2010) and with Bayesian cluster analysis (Structure 2.3.3; Pritchard et al. 2000). For the Bayesian analysis, we tested *K* = 2–4 genetic clusters, which each were run 20 times, 20,000 burn-in and 50,000 generations per run. We judged the genetic difference between “wrong” host-plant individuals (2) and survival alleles (3) by comparing the membership value of the random population to the proportion of individuals with membership values >50% relative to outliers that should belong to the alternative host race.

In the third analysis, we studied associations between survival and genotypes by comparing the genetic membership of individuals surviving the shortest and the longest on alternative plants with individuals sampled randomly on their own host plants (Fig. 1). The difference between the random sample and the short- and long-survival samples *within* host races can be used to differentiate between divergence *between* host races caused by “survival alleles” or “host-race alleles” of other significance. If alleles/loci are related to survival, they should ideally be at low frequency in the short-survival sample, intermediate in the random own plant sample, and high frequency in the random alternative plant sample. For example, “survival alleles” are indicated when the genetic makeup of nettle animals surviving long on field bindweed (the wrong host) is correlated with that of a random sample of bindweed animals from field bindweed (i.e. they are “bindweed-like”) rather than the random nettle sample. For each host race, we tested for genetic correlations by genotyping 10 males and 10 females (total *N* = 20) in six classes (Fig. 1). To avoid cage effects, each of the 12 groups (2 sexes × 6 classes) were made up of one individual per cage, with a maximum of two individuals from one cage when needed to get sufficient sample numbers. The genetic assignment was estimated with Bayesian clustering analyses (Structure 2.3.3), fixing *K* = 2. We also tested whether individual loci were related to survival, studying only alleles with frequencies >0.10 in the total test sample (*N* = 124). Differences in allele frequencies among survival classes were quantified with ANOVA. The DNA protocol and PCR conditions are described in the study of Maniyar et al. (2013).

In a post hoc test, we assessed the genetic host-race affiliation of seven nettle animals that tested positive for stolbur tuf-b (i.e., field bindweed stolbur, see Results) using Structure 2.3.3. The genetic memberships of the seven animals were compared with memberships from the “survival” data set above with fixed *K* = 2.

## Results

### Stolbur infection rates

Stolbur infection rates differed significantly between the host races, *P* < 0.001, bindweed = 0.28 (*N* = 627), nettle = 0.15 (*N* = 591) but did not differ significantly between the sexes in each host race, bindweed: *P* = 0.10 (*N*_female_ = 312 (96 infected), *N*_male_ = 315 (81 infected)), nettle: *P* = 0.79 (*N*_female_ = 302 (43 infected), *N*_male_ = 289 (47 infected) (Fisher's exact tests). All 177 infected bindweed animals were infected with tuf-b. Of the 90 infected nettle animals, 83 were infected with tuf-a, while seven individuals (three males and four females) were infected with tuf-b. Because tuf-b is associated with field bindweed, the tuf-b positive nettle animals were removed from tests for effects of stolbur infection in nettle animals.

### Survival – effect of sex and week

The twice repeated experiments showed that males in both host races survived significantly longer than females on the own host plant but that the difference between the sexes was less or disappeared on the alternative host plant (Table 1). The lack of difference between the sexes on the alternative plant was thus caused by higher relative mortality of males (mean of four comparisons = 0.57) on the “wrong” host plant than females (0.37).

**Table 1:** Mean survival in days between males and females of *Hyalesthes obsoletus* host races in two experimental weeks (1 and 2). Males survived significantly longer than females on own host plants but not on the alternative plant. Numbers in brackets indicate sample sizes. Infection status of plants: S = symptomatic field bindweed, N = nonsymptomatic field bindweed. Kaplan–Meier survival analysis, Chi-squared distributed with 1 degree of freedom.

Host plant	Recipient plant	Symptoms	Week 1				Week 2			
			Male	Female	χ^2^	*P*	Male	Female	χ^2^	*P*
Bindweed	Bindweed	N	6.75 (58)	5.46 (54)	9.3	**	6.94 (50)	3.62 (50)	29.4	***
		S	7.31 (54)	4.98 (46)	17.3	***	10.07 (44)	5.31 (55)	22.7	***
Bindweed	Nettle		4.66 (59)	3.68 (57)	4.4	*	3.28 (50)	3.60 (50)	0.9	ns
Nettle	Nettle		13.64 (50)	10.42 (50)	3.7	0.05	10.14 (50)	4.96 (50)	19.7	***
Nettle	Bindweed	N	3.34 (41)	3.33 (55)	0.1	ns	2.86 (50)	2.76 (50)	0.2	ns
		S	3.31 (49)	3.41 (46)	0.3	ns	2.96 (49)	2.64 (51)	3.2	ns

ns, not significant.

* *P* < 0.05

** *P* < 0.01

*** *P* < 0.001.

The repeated experiments also showed that nettle animals of both sexes on average lived longer in the first week (Table 2), independent of plant treatment. For bindweed animals, the results were heterogeneous with significantly lower survival in two comparisons and increased survival in two other of which one was significantly, *P* < 0.05, increased. The cause for the latter result was associated with increased survival on symptomatic field bindweed in the second week (see below, Table 3).

**Table 2:** Mean survival in days of *Hyalesthes obsoletus* host races in two experimental weeks (1 and 2). Nettle animals always survived longer in week 1, whereas the effect is less pronounced in bindweed animals. The significant increase in survival of bindweed males in week 2 – opposite to the general pattern – was caused by prolonged survival on symptomatic field bindweed (see Table 3). Sample sizes: *N*_1_ = sample size in first week, *N*_2_ = sample size in second week. Infection status of field bindweed: S = symptomatic, N = nonsymptomatic. For nettle animals on field bindweed, the N and S treatments were pooled due to no plant-infection effect. Kaplan–Meier survival analysis, Chi-squared distributed with 1 degree of freedom.

Host plant	Recipient plant	Symptoms	Sex	*N*_1_/*N*_2_	Experiment		χ^2^	*P*
					Week 1	Week 2		
Bindweed	Bindweed	N	M	58/50	6.75	6.94	0.2	ns
		N	F	54/50	5.46	3.62	26.8	***
		S	M	54/44	7.31	10.07	13.3	***
		S	F	46/55	4.98	5.31	0.4	ns
Bindweed	Nettle		M	59/50	4.66	3.28	9.9	**
			F	57/50	3.68	3.60	0.2	ns
Nettle	Nettle		M	50/50	13.64	10.14	4.9	*
			F	50/50	10.40	4.96	17.4	***
Nettle	Bindweed	N & S	M	90/99	3.32	2.91	7.6	**
		N & S	F	101/101	3.36	2.70	12.3	***

ns, not significant.

* *P* < 0.05

** *P* < 0.01

*** *P* < 0.001.

**Table 3:** Mean survival in days of *Hyalesthes obsoletus* collected on field bindweed and stinging nettle exposed to own and alternative plants, and in relation to stolbur infection, in two identical experiments (Week 1 & 2). Bindweed-S and Bindweed-N refer to stolbur symptomatic and nonsymptomatic field bindweed, respectively. Ho-I and Ho-N refer to stolbur-infected and noninfected *H. obsoletus*. M = males, F = females. Numbers in brackets indicate sample sizes. Kaplan–Meier survival analysis, Chi-squared distributed with 1 degree of freedom.

Origin	Week	Sex	Recipient plant								Stolbur infection of *H. obsoletus* relative to recipient plant							
			Bindweed^1^	Nettle	χ^2^	*P*	Bindweed-N	Bindweed-S	χ^2^	P	Ho-N Bindweed	Ho-I Bindweed	χ^2^	P	Ho-N Nettle	Ho-I Nettle	χ^2^	*P*
Bindweed	1	M	7.03 (112)	4.66 (59)	24.9	***	6.75 (58)	7.31 (54)	1.1	ns	7.08 (83)	6.86 (29)	0.3	ns	4.91 (46)	3.77 (13)	1.8	ns
		F	5.24 (100)	3.68 (57)	27.5	***	5.46 (54)	4.98 (46)	0.5	ns	5.43 (65)	4.89 (35)	0.9	ns	3.76 (42)	3.47 (15)	0.4	ns
	2	M	8.40 (94)	3.28 (50)	75.6	***	6.94 (50)	10.07 (44)	9.5	**	8.05 (74)	9.70 (20)	1.7	ns	3.19 (31)	3.42 (19)	0.3	ns
		F	4.50 (105)	3.60 (50)	5.1	*	3.62 (50)	5.31 (55)	10.2	**	4.60 (78)	4.22 (27)	0.2	ns	3.61 (31)	3.58 (19)	0.1	ns
Nettle	1	M	3.32 (90)	13.64 (50)	61.7	***	3.34 (41)	3.31 (49)	0.1	ns	3.37 (83)	2.71 (7)	0.3	ns	12.60 (42)	19.13 (8)	0.9	ns
		F	3.37 (101)	10.42 (50)	44.9	***	3.33 (55)	3.41 (46)	0.1	ns	3.37 (91)	3.37 (8)	0.1	ns	10.07 (45)	13.60 (5)	0.1	ns
	2	M	2.91 (99)	10.14 (50)	54.1	***	2.86 (50)	2.96 (49)	1.2	ns	2.95 (79)	2.75 (19)	1.5	ns	10.16 (38)	11.00 (10)	0.2	ns
		F	2.70 (101)	4.96 (50)	26.5	***	2.76 (50)	2.65 (51)	0.6	ns	2.73 (84)	2.63 (16)	0.2	ns	4.64 (39)	6.40 (10)	0.5	ns

ns, not significant.

* *P* < 0.05

** *P* < 0.01

*** *P* < 0.001.

1 Includes symptomatic and nonsymptomatic plants.

### Survival – effects of host plant and stolbur infection

Due to the overall significant effects of sex and experimental week, the effects of host plant and stolbur infection on survival were analyzed separately for each sex and week.

During the daily visual observations, the insects were frequently found inserting their stylets, thus confirming their feeding activity on the host plants. Both sexes of host races lived significantly longer on own than on alternative plants. The result was consistent between weeks (Table 3). The only significant effect of plant symptoms was observed for bindweed males and females in the second week, where survival was significantly higher on symptomatic than on nonsymptomatic field bindweed. There were no significant effects of stolbur infection in *H. obsoletus* in either host race. However, mean survival of infected nettle animals on stinging nettle was in three instances much higher than for noninfected animals, but the low infection rate of 0.15 prohibited a rigorous test size.

Nettle animals survived significantly longer on nettle than bindweed animals on field bindweed, and bindweed animals on stinging nettle survived significantly longer than nettle animals on field bindweed (Table 4). Hence, nettle animals were significantly more negatively affected by the transfer to field bindweed than bindweed animals to stinging nettle. This was found both for direct comparisons of mean surviving days on alternative plants (Table 4) as well as for relative survival on own and alternative plants (Table 5). Mean relative survival of nettle animals on field bindweed (0.345) was significantly lower than for bindweed animals on stinging nettle (0.705), two-tailed t-test *P* = 0.025, *t* = −2.96, df = 6.

**Table 4:** Comparisons of survival on alternative plants. Nettle animal survive longer on stinging nettle (N on N) than bindweed animals on field bindweed (B on B), and bindweed animals survived on average approximately 1 day longer on stinging nettle (B on N) than nettle animals transferred to field bindweed (N on B). Only bindweed animals on nonsymptomatic field bindweed included. Significances were estimated with Kaplan–Meier survival analyses; all tests are Chi-squared distributed with 1 degree of freedom.

Sex	Week	N on B	B on N	*χ*^2^	*P*	N on N	B on B	*χ*^2^	*P*
Male	1	3.34	4.66	8.7	**	13.64	6.76	23.0	***
Male	2	2.86	3.28	5.5	*	10.14	6.94	6.2	*
Female	1	3.33	3.68	1.5	ns	10.42	5.46	14.2	***
Female	2	2.76	3.60	12.7	***	4.96	3.62	4.1	*

ns, not significant.

* *P* < 0.05

** *P* < 0.01

*** *P* < 0.001.

**Table 5:** Relative survival on alternative plant relative to own plant. Mean survival of nettle animals on field bindweed (N on B) relative to nettle animals on stinging nettle (N on N) was significantly lower than mean survival of bindweed animals on stinging nettle (B on N) relative to bindweed animals on field bindweed (B on B), two-tailed t-test *P* = 0.025, *t* = −2.96, df = 6. The relationships are based on estimates presented in Table 4.

Sex	Week	N on B/N on N	B on N/B on B
Male	1	0.24	0.69
Male	2	0.31	0.47
Female	1	0.32	0.67
Female	2	0.51	0.99

In summary, survival in relation to nonsymptomatic host plants followed a hierarchy where survival of nettle animals on stinging nettle > bindweed animals on field bindweed > bindweed animals on stinging nettle > nettle animals on field bindweed, and where a transfer to the alternative plant affected males relatively more negatively than females.

### Genetic differentiation

In a first analysis, we tested the genetic diversification between field bindweed and nettle animals to assess their host-race status. Structure analysis of the two random experimental samples and two distant syntopic bindweed and nettle populations identified *K* = 2 genetic clusters (mean 20 runs, *K* = 2: −ln = 1915.62 ± 2.99, *K* = 3: −ln = 1962.09 ± 30.24, *K* = 4: −ln = 1964.42 ± 17.90) as the most likely, where the samples clustered according to plant but not location (Appendix S1). There was no effect of sex, thus sexes were pooled in further analyses. The mean proportion of membership to each of the two experimental plant populations (random samples) (bindweed = 0.72 nettle = 0.69) indicated clear divisions but was lower than the proportion of membership for the distant syntopic populations (memberships = 0.86 and 0.89). The pairwise differentiation between the two distant bindweed populations, *F*_ST_ = 0.020 (*P* = 0.05), and two distant nettle populations, *F*_ST_ = 0.012 (*P* = 0.14), was a third to half of that between the experimental bindweed and nettle populations, *F*_ST_ = 0.038 (*P* < 0.001). The mean expected heterozygosity of the bindweed population, *H*_e_ = 0.78, was slightly greater than in the nettle population, *H*_e_ = 0.75, as observed throughout the host-race range (Imo et al. 2013).

In the second analysis, we judged the possibility that some individuals originated from the alternative plant, despite that all individuals were caught on the own plant, as both host plants are common and occur in the investigated area. Comparisons of the individual membership scores indicated high variability among individuals in both host races, but in only one instance (of 124), did we observe an outlier membership to the alternative plant in a bindweed animal (Appendix S2). The seven nettle-individuals with field bindweed associated tuf-b stolbur belonged genetically to nettle (nettle probability scores: 0.61–0.95), whereas one was more bindweed-like (bindweed probability score: 0.77). We therefore conclude that the individuals overwhelmingly belonged to the anticipated host race.

In the third analysis, we analyzed survival on alternative plants relative to microsatellite multilocus genotypes with Structure. For both host races, the group of individuals surviving long on the alternative plant was on average less related to the own population (membership score: bindweed animals, 0.808; nettle animals, 0.806) than the group of individuals surviving short (0.874 and 0.830), that is, in accordance with predicted positive gene-survival correlations for survival. However, both the short-survival and the long-survival groups of each host race were more related to the own plant population than a random sample from the own plant population (0.784 and 0.748). Hence, the signals of gene-survival correlations in the long- and short-survival groups were likely coincidental, and we conclude that there were no survival relationships with the seven microsatellite loci (Appendix S2). Subsequent analyses for correlations between allele frequencies from individual loci with survival also failed to find any associations (results not shown).

## Discussion

The evolution of insect-vectorborne plant diseases is tied to the niche breadth of the insect vector and its ability to acquire and transmit pathogens among host plants (Grilli and Holt 2000; Lopes et al. 2009). Hence, insect-vectorborne epidemiology is intimately associated with the evolution of insect/plant interactions. A key question is whether vectored endosymbionts influence the vector's adaptation to different host plants (Mayer et al. 2008; Biere and Tack 2013). In the present study, we studied the dynamics of an important fitness parameter (adult survival) as a proxy for adaptive traits in ecological host-race specialization and whether infection by an obligate vectored pathogen/symbiont could be related to the specialization process.

Our reciprocal survival experiments showed not only that absolute but also relative adult survival of both host races was highest on own plants, indicating that adult survival in the host-race populations has an adaptive component. The experiments also showed an asymmetric survivorship between the host races where field bindweed, the original plant, was less suitable for nettle animals, the novel host race, than vice versa. This might be caused in part by stinging nettle twigs being more nutritious or holding more fluid, but because bindweed animals survived longer on field bindweed twigs than they did on stinging nettle twigs, the host-plant effect is stronger than the phenotypic “twig-effect.”

In contrast to an adaptive effect of host plant, we found only circumstantial evidence for an effect of stolbur infection on the vector's survival potential. The consequences of being infected with stolbur indicate a neutral to positive rather than a negative effect: No difference in survival of infected and noninfected bindweed animals on field bindweed and longer, though not significant, survival of infected nettle animals on stinging nettle. The significantly longer survival of bindweed animals on symptomatic field bindweed in the second experiment is suggestive for a fostering effect of plant infection, but due to the lack of phytoplasma detection in plants other effects (e.g., physiological) cannot be excluded. Conversely, there was no increased negative effect of infection when vectors were subjected to an alternative host plant, that is, infected with the “wrong” stolbur strain. No effect of stolbur infestation on the vector has also been found for nymphal development in nettle animals (Kaul et al. 2009). These observations indicate again that the host plant is the dominant cause for adult survival and imply adaptation for stolbur phytoplasma in general rather than for specific and divergent strains. The absence of strain effects was also observed in *Euscelidius variegatus* infected with Flavescence-dorée phytoplasma, which however showed a negative effect on the fitness of its vector (Bressan et al. 2005).

The comparison of the two experimental vector populations and two distant sympatric populations revealed two genetic clusters according to the host plant but not location, confirming the results of Imo et al. (2013). As the genetic host-race pattern is consistent throughout the host-race range and the plants are ubiquitous, each with intraspecifically consistent phenology, there is little supposition for local adaptation overriding plant-related adaptation for survival. We further did not observe correlations between survival and genetic diversification at seven microsatellite loci, which each contributes to the genetic distinctness of the two host races. This finding corroborates the geographic distribution of microsatellite allele frequencies, which suggest that genetic drift during a single founding event is responsible for diversification at these loci (Imo et al. 2013).

### Survival and mating isolation

A universal resource parameter in host-race evolution is host-plant phenology, which creates allochronic flight and mating times (Mercader et al. 2009; Loxdale 2010). Temperature sums for emergence of *H. obsoletus* differ significantly between host races. In Germany, the peak seasonal flight overlap is 1–2 weeks (Maixner and Johannesen in press[Bibr b38]). By contrast, flight overlap in more southern regions is longer, up to 4 weeks (Forte et al. 2010). In our study, the mean survival on alternative hosts was significantly reduced to a time span (2–3 days) that may not be long enough for oviposition. The low survival on alternative hosts might even suggest that when suddenly exposed to the wrong host most individuals may not feed at all, a behavior observed in sympatric host races of pea aphids (Caillaud and Via 2000). In the field, this behavior would translate to evolving complete avoidance of the alternative plant. Experiments studying tactile attraction to the two host plants (Imo 2013) indicate clear preferences for the natal plants. Our results further showed that the transfer effect is significantly greater in males than in females (see also Calcagno et al. 2007). As males initiate mating by displaying (calling) for females on host plants (Mazzoni et al. 2010), males should avoid suboptimal plants as mating locations. Because females move much less than males among host plants (Maixner and Johannesen 2012) male plant choice, also in the sense of plant avoidance, and female mate choice may lead to positive assortative mating, a fundamental assumption for reproductive isolation in host-race models of sympatric speciation (Dieckmann and Doebeli 1999; Coyne and Orr 2004). Host (plant) avoidance, not only attraction, has been put forward as a significant but overlooked isolation mechanism (Forbes et al. 2005; Feder and Forbes 2007).

### Survival and specialization versus plasticity

The observed asymmetry in survival between the host races might be expected in polyphagous species where the ancestral, polyphagous population has a broader niche breadth than the evolved and specialized population. However, this notion may be confounded by preference/acceptance hierarchies, which may trigger nonhereditary preferences/performance correlations (Singer et al. 1992), or by genetically specialized local populations that are perceived as polyphagous in the total distribution range (Loxdale et al. 2011). Indeed, stinging nettle and field bindweed are common plants throughout the distribution range of *H. obsoletus,* but feeding behavior in *H. obsoletus* is tied in geographic preference hierarchies (Mori et al. 2008; Kessler et al. 2011). It is therefore of fundamental interest to discern whether trait evolution in newly formed host race exceeds plasticity in general (Loxdale et al. 2011).

Kessler et al. (2011) analyzed survival and host preferences of nymphs and adults originating from stinging nettle in southwest Switzerland where it is a highly preferred plant, while field bindweed is a very rare host. Southwest Swiss populations associated with stinging nettle and field bindweed cannot be differentiated genetically (Maniyar et al. 2013), but they are genetically divergent from German nettle and bindweed host-race populations (Johannesen et al. 2012; Imo et al. 2013). Both nymphs and adults of nettle animals survived longer on stinging nettle, but nymphs had no inherent host-plant preferences. Microsatellite data and stolbur infections of *H. obsoletus* both indicate that in Switzerland adults from stinging nettle move between stinging nettle and field bindweed (Maniyar et al. 2013). Field bindweed is likely used as an alternative, secondary host plant. In contrast to the nettle population studied by Kessler et al. (2011), the situation in Germany is such that field bindweed was the common host of *H. obsoletus* prior to the recent inclusion of stinging nettle. Thus, the two nettle-associations differ in their historical, genetic and resource backgrounds but produced similar adaptive implications for the trait survival.

Given that both host plants are common and co-occur, the two studies combined implicate the use of “general purpose” plants but also that given the right environmental conditions, polyphagous species such as *H. obsoletus* have the potential to exceed innate plasticity and specialize. The conjecture made by Singer (1982) that host preference may not change when host shifts are caused by the scarcity of preferred hosts, oppositely assumes that preference should evolve in sympatry when both hosts are common. The latter is the case in *H. obsoletus* for which host races show clear tactile preferences but only the ancestral race, bindweed animals, shows volatile preferences (Imo 2013). Hence, the new race has evolved partial avoidance (specialization) only. Our results contrast other studies of host-race formation in polyphagous species where preference but not performance is lost during host-race evolution (Ohshima 2008). Today, the two host races also differ in acoustic signals (S. Grube pers. comm.), as do *Wolbachia* infection rates (Johannesen et al. 2009). Such suits of fitness-related phenotypic differences between host races have been found in several species that have diverged in historical times and indicate that host-race evolution affects many traits simultaneously and can proceed extremely fast (Carroll et al. 2001; Loxdale 2010).

## Data Accessibility

Excel table with individual survival data: DRYAD entry doi: 10.5061/dryad.dh05q Data file: DRYAD_Survival.

Structure file with microsatellite data for genotype-survival correlations: DRYAD entry doi: 10.5061/dryad.dh05q Data file: DRYAD_genotypes.

## Supporting Information

Appendix S1 Genetic membership proportions of *H. obsoletus* associated field bindweed and stinging nettle populations (distance: 7 km) used for survival experiments compared with geographically distant (ca. 100 km) syntopic host-plant populations. Geographically distant populations associated with the same plant are more related than geographically close populations from different plants.

Appendix S2 Genetic membership proportions of a group of *H. obsoletus* surviving short and long on alternative plants and of a random group from the own plant.
