# Supplementary material for: Survival relative to new and ancestral host plants, phytoplasma infection, and genetic constitution in host races of a polyphagous insect disease vector
Source: Ecol Evol. 2014 Jul 15;4(15):3082–92. doi: 10.1002/ece3.1158 (PMC4161181; doi:10.1002/ece3.1158)
Supplement: Appendix S1 — Genetic membership proportions of H. obsoletus associated field bindweed and stinging nettle populations (distance: 7 km) used for survival experiments compared with geographically distant (ca. 100 km) syntopic host-plant populations. Geographically distant populations associated with the same plant are more related than geographically close populations from different plants. [file ece30004-3082-sd1.ppt]

## Slide 1
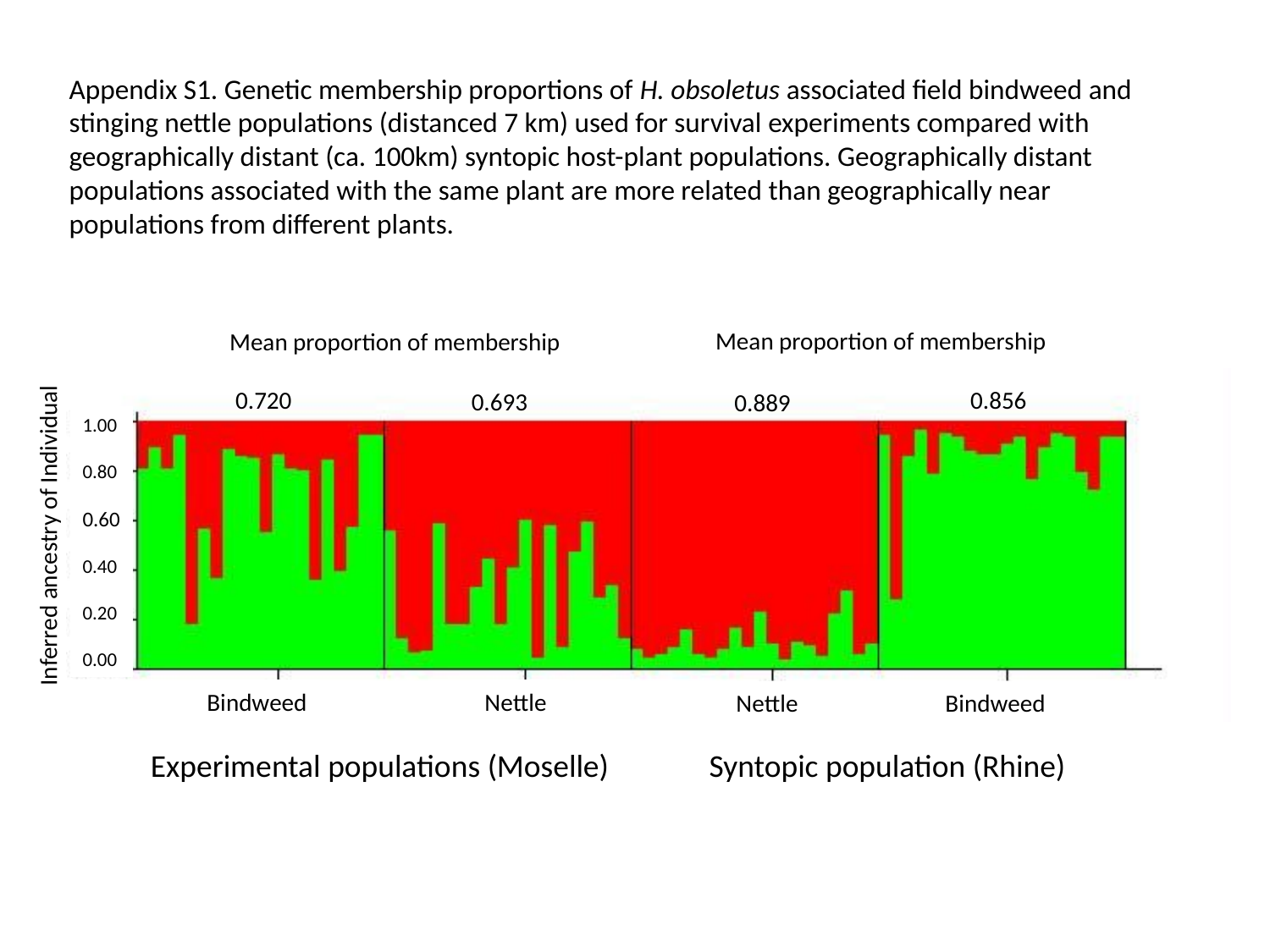

Appendix S1. Genetic membership proportions of H. obsoletus associated field bindweed and stinging nettle populations (distanced 7 km) used for survival experiments compared with geographically distant (ca. 100km) syntopic host-plant populations. Geographically distant populations associated with the same plant are more related than geographically near populations from different plants.
Mean proportion of membership
Mean proportion of membership
0.720
0.856
0.693
0.889
1.00
0.80
0.60
0.40
0.20
0.00
Inferred ancestry of Individual
Bindweed
Nettle
Nettle
Bindweed
Experimental populations (Moselle)
Syntopic population (Rhine)
