# Supplementary material for: Survival relative to new and ancestral host plants, phytoplasma infection, and genetic constitution in host races of a polyphagous insect disease vector
Source: Ecol Evol. 2014 Jul 15;4(15):3082–92. doi: 10.1002/ece3.1158 (PMC4161181; doi:10.1002/ece3.1158)
Supplement: Appendix S2 — Genetic membership proportions of a group of H. obsoletus surviving short and long on alternative plants and of a random group from the own plant. [file ece30004-3082-sd2.ppt]

## Slide 1
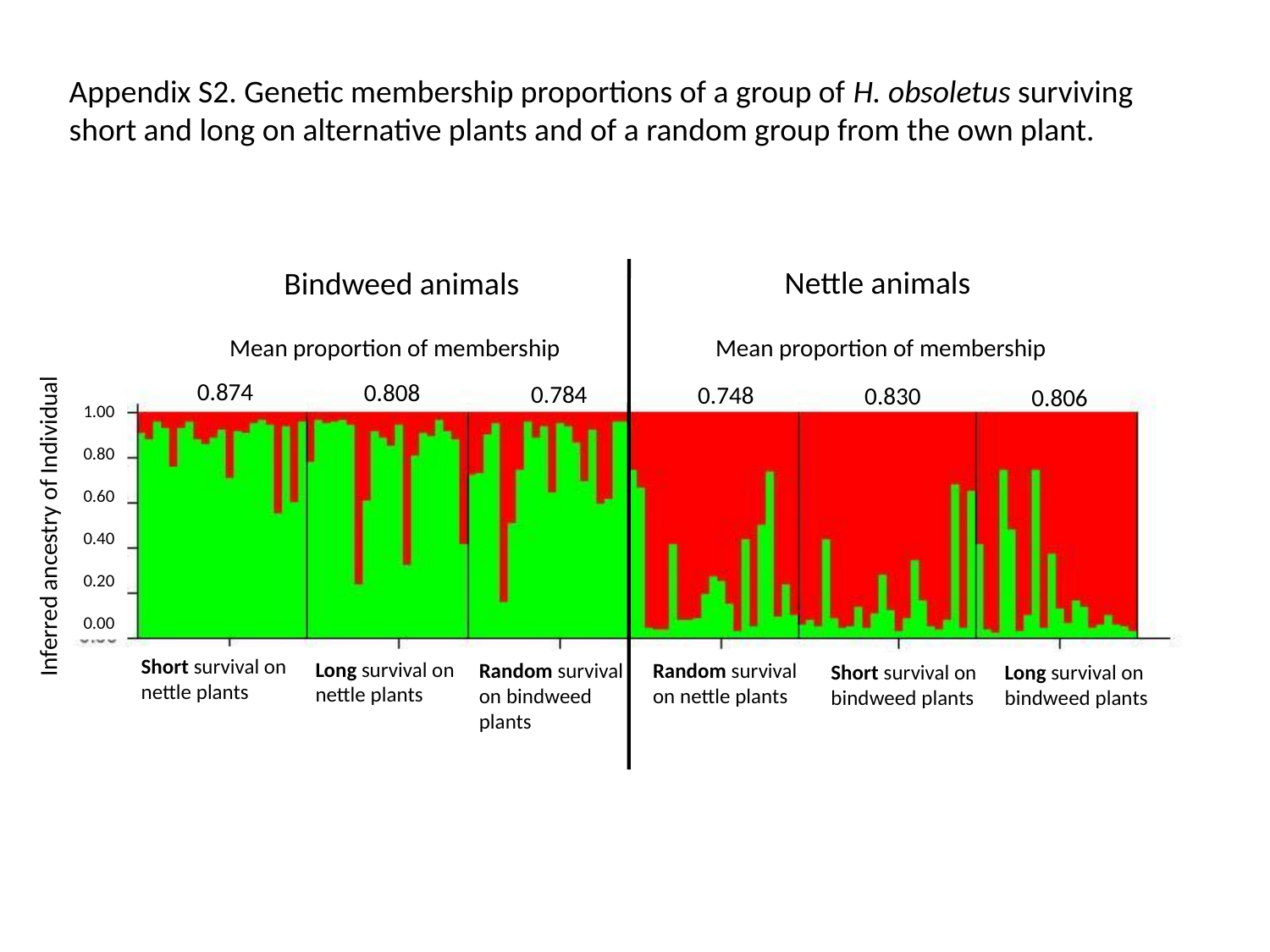

Appendix S2. Genetic membership proportions of a group of H. obsoletus surviving short and long on alternative plants and of a random group from the own plant.
Nettle animals
Bindweed animals
Mean proportion of membership
Mean proportion of membership
0.874
0.808
0.784
0.748
0.830
0.806
1.00
0.80
0.60
0.40
0.20
0.00
Inferred ancestry of Individual
Short survival on nettle plants
Long survival on nettle plants
Random survival on nettle plants
Random survival on bindweed plants
Short survival on bindweed plants
Long survival on bindweed plants
